# Bleeding risks with novel oral anticoagulants especially rivaroxaban versus aspirin: a meta-analysis

**DOI:** 10.1186/s12959-021-00322-6

**Published:** 2021-10-02

**Authors:** Xiehui Chen, Weichao Huang, Aimei Sun, Lili Wang, Fanrui Mo, Wenqin Guo

**Affiliations:** 1 Department of Cardiology, Shenzhen Longhua District Central Hospital, No. 187, Guanlan Road, Longhua District, Shenzhen, China; 2 Department of Cardiology, Fuwai Hospital Chinese Academy of Medical Sciences, No. 12, Langshan Road, Nanshan District, Shenzhen, China

**Keywords:** Anticoagulants, Aspirin, Intracranial hemorrhages, Gastrointestinal hemorrhage

## Abstract

**Background:**

This pairwise meta-analysis determines the difference in bleeding risks associated with the use of novel oral anticoagulants (NOACs) and aspirin.

**Methods:**

PubMed, the Cochrane Library database, clinicaltrial.gov, and related studies were searched for randomized control trials (RCTs) comparing NOAC and aspirin published between January 1, 2000 and May 10, 2021. The primary endpoint was intracranial hemorrhage (ICH).

**Results:**

Eleven studies involving 57,645 patients were included. Compared to aspirin, rivaroxaban (5 mg/day) had a similar risk of ICH, major bleeding, and fatal bleeding; rivaroxaban (10 mg/day) had higher risks of gastrointestinal hemorrhage (OR: 1.41; 95% CI: 1.03–1.94; *P* = 0.032; I^2^ = 0%) and a similar risk of ICH, major bleeding, and fatal bleeding; and rivaroxaban (15–20 mg/day) had higher risks of ICH (OR: 3.21; 95% CI: 1.36–7.60; *P* = 0.008; I^2^ = 0%), major bleeding (OR: 2.64; 95% CI: 1.68–4.16; *P* < 0.001; I^2^ = 0%), and fatal bleeding (OR: 2.26; 95% CI: 1.25–4.08; *P* = 0.007; I^2^ = 0%) and a similar risk of gastrointestinal hemorrhage. Bleeding outcomes between other NOACs (apixaban and dabigatran etexilate) and aspirin were not different.

**Conclusions:**

The bleeding risks associated with NOACs depend on drug type and dosage. For ≥15 mg/day of rivaroxaban, the risk of ICH was significantly higher than that with aspirin. However, further studies comparing dabigatran etexilate and apixaban versus aspirin are warranted to draw a definite conclusion.

**Supplementary Information:**

The online version contains supplementary material available at 10.1186/s12959-021-00322-6.

## Background

Compared with the traditional anticoagulant warfarin, novel oral anticoagulants (NOACs) have several advantages, including being less affected by food or drugs and not requiring frequent monitoring of the international normalized ratio [1]. The efficacy of NOACs at preventing ischemic strokes is not inferior to that of warfarin, and NOACs are associated with a lower risk of intracranial hemorrhage (ICH) than warfarin in patients with high-risk atrial fibrillation (AF) [2]. NOACs are also recommended for the treatment of coronary heart disease (CHD) and venous thromboembolism (VTE) [3, 4].

However, the bleeding profile of NOACs when used in clinical practice remains controversial. The NAVIGATE ESUS, a randomized controlled trial (RCT) evaluating the benefits of rivaroxaban for patients with embolic strokes of undetermined source (ESUS), found that rivaroxaban (10 mg/day) did not reduce the rate of strokes (ischemic or hemorrhagic) or systemic emboli, but increased the risk of ICH compared to aspirin (100 mg/day) [5]. The COMPASS study reported that the rate of ICH and gastrointestinal bleeding in patients administered rivaroxaban (10 mg/day) was higher than that in patients administered aspirin (100 mg/day) [6]. However, other studies have reported that the bleeding risks of NOACs are similar to those of aspirin [7–9]. Therefore, it is unclear if NOACs are associated with a higher bleeding risk than aspirin. Studies regarding the use of NOACs for other cardiovascular diseases are currently being conducted, including one study comparing the efficacy of NOACs and aspirin for the treatment of patients with CHD and gastrointestinal diseases undergoing percutaneous coronary intervention [10].

In most of the previously published anticoagulant studies, bleeding was the secondary endpoint, suggesting that the statistical power of these studies was not sufficient to assess the bleeding risk of anticoagulants. A meta-analysis, which can gather all current evidence thereby expanding the sample size and improve the accuracy of estimated outcomes, is a common methodology for the evaluation of a drug’s safety profile [11, 12]. This meta-analysis aimed to compare the bleeding risks associated with NOAC use to those of aspirin to guide both clinical practice and research regarding the selection of NOAC type and dosage.

## Methods

This study was conducted according to the Preferred Reporting Items for Systematic Reviews and Meta-Analyses (PRISMA) Statement [13]. PubMed, the Cochrane Library database, clinicaltrial.gov, and the references of related studies were searched by two researchers (Lili Wang and Aimei Sun) for studies published between January 1, 2000 and May 10, 2021. The following keywords and MeSH terms were used: “NOAC,” “new oral anticoagulant,” “direct oral anticoagulant,” “non-vitamin K oral anticoagulant,” “rivaroxaban,” “dabigatran etexilate,” “apixaban,” “edoxaban.” The research strategies are shown in Supplemental Appendix.

### Inclusion and exclusion criteria

RCTs which had an intervention group that received NOACs (rivaroxaban, dabigatran etexilate, apixaban, or edoxaban) and a control group that received aspirin, which reported one of the clinical outcomes of interest of this study, and which were published in the English language were included in this meta-analysis. Studies in which the intervention group included other anticoagulants (such as warfarin or heparin), the control group was not administered aspirin, the study design was observational or cohort, the outcomes of interest were not reported, or the language of reporting was not English were excluded from this meta-analysis.

### Outcomes

The primary endpoint of this meta-analysis was ICH. The secondary endpoints included fatal bleeding, major bleeding, and gastrointestinal hemorrhage. ICH, fatal bleeding, and gastrointestinal hemorrhage were defined similarly among the included studies, though major bleeding was not. The definitions of the clinical outcomes in the included studies are shown in Supplemental Table 1. The definition of major bleeding in most of the studies was based on the one that is proposed by the International Society on Thrombosis and Haemostasis [14]. The definition of ICH was traumatic and atraumatic intracerebral, subarachnoid, and subdural or epidural hemorrhage (does not include microbleeds or hemorrhagic transformation, does include intraspinal).

### Data extraction and study quality assessment

Two researchers (Weichao Huang and Wenqin Guo) independently extracted the year of publication, mean follow-up time, dosages of the intervention and control groups, total number of events and patients in each group, indications for antithrombotic drugs, and characteristics of the included studies (such as the average age and proportions of stroke, hypertension, and diabetes mellitus). When multiple reports for the same clinical trial were identified, data from the most recent report were used. When the extracted data differed between the two researchers, a third researcher helped us reach a final decision. The intention-to-treat sample size was used for analysis, and the quality of the included studies was assessed by two researchers (Lili Wang and Fanrui Mo) using the Cochrane risk-of-bias tool [15]. If there was a disagreement between the two researchers, the third researcher (Wenqin Guo) made the decision.

### Statistical analysis

The odds ratio (OR) and the corresponding 95% confidence interval (CI) were used as measures of the effect size. –The random effects model was used for meta-analysis (Mantel-Haenszel method) because the population characteristics were different between the studies [16]. Cochrane Q tests and the inconsistency index (I^2^ test) were used to assess the statistical heterogeneity among the included studies [17]. An I^2^ value < 25% indicated the absence of heterogeneity, an I^2^ value between 25 and 50% indicated low heterogeneity, an I^2^ value between 50 and 75% indicated moderate heterogeneity, and an I^2^ value > 75% indicated high heterogeneity [17]. The funnel plot and Egger’s regression asymmetry test were used to assess for publication bias [18]. Subgroup analyses were conducted based on the indications for antithrombotic medications (AF, ESUS, CHE, or VTE); the 12-month incidence of ICH in patients with ischemic stroke is 15 times higher than that in patients without ischemic stroke, and AF patients who are not treated with a vitamin K antagonists are at a higher risk of bleeding [19, 20]. Statistical significance was set at *P* < 0.05. The meta-analysis was conducted using STATA Software Version 12.0, (StataCorp, University City, Texas, USA) and Review Manager Software (version 5.4, The Cochrane Collaboration, Copenhagen, Denmark).

## Results

**Fig. 1:**
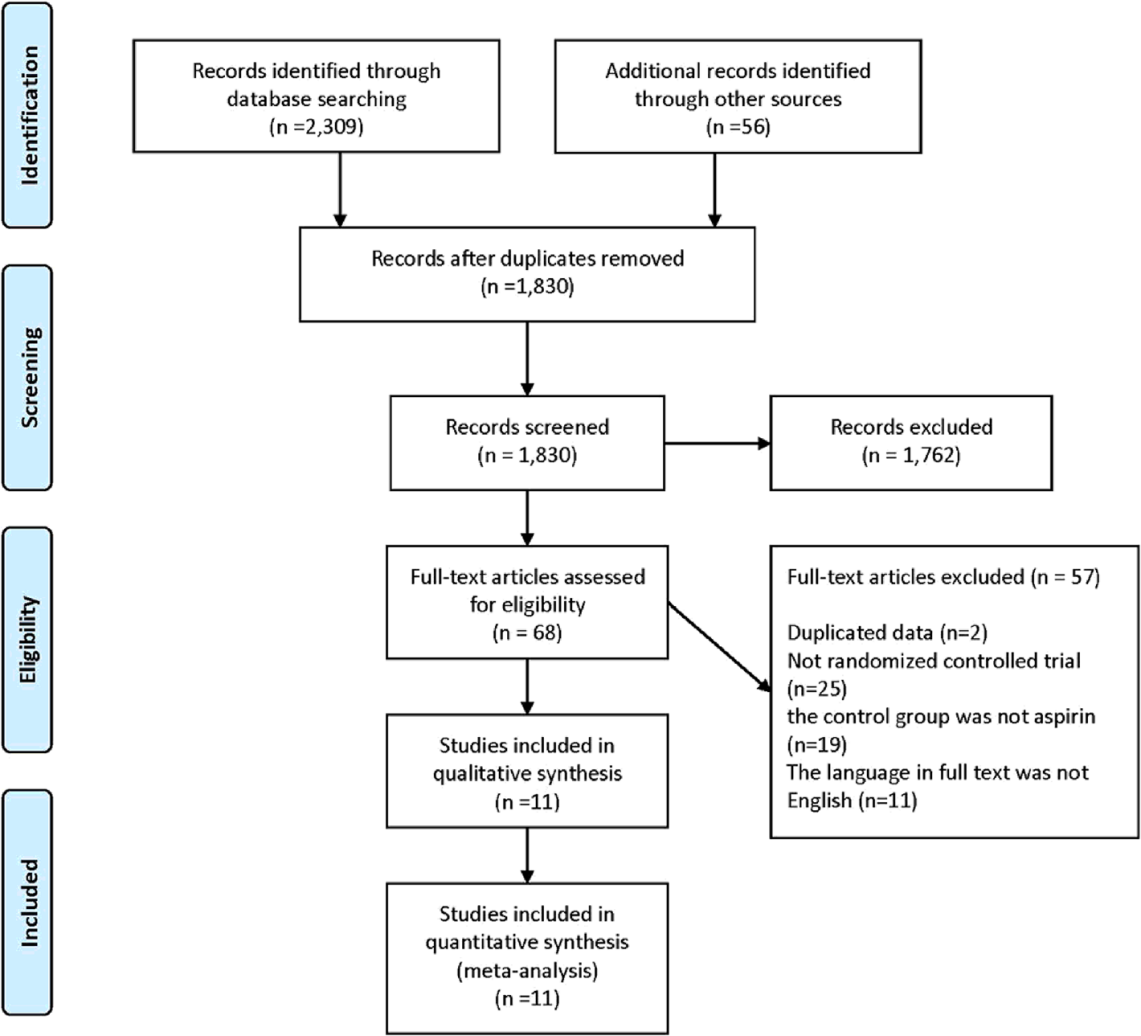
The flow chart of literature search and selection

**Fig. 2:**
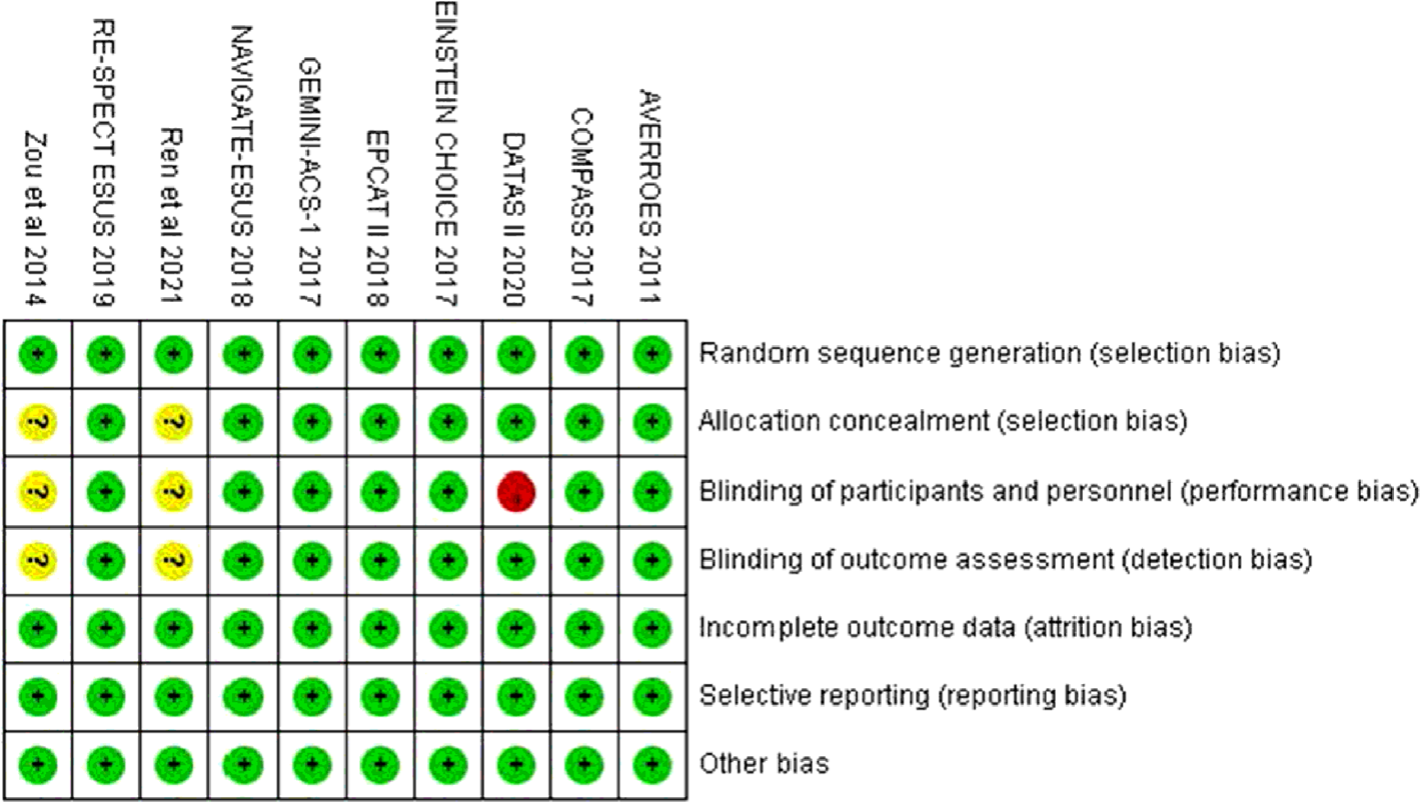
The assessment of Cochrane risk-of-bias

During screening, abstracts of 1830 studies were read (Fig. 1). Eleven studies reporting 10 RCTs involving 57,645 patients were included in this analysis [5–9, 21–26]. A study conducted by Anand et al. [27] was excluded as it included patients with stable peripheral or carotid artery disease whose data were included in the study by Eikelboom et al. [6]. The GALILEO study [28] and the ePAD trial [29] were excluded because some or all of the control groups received clopidogrel. A study by Zou et al. included three treatment arms (aspirin, low-molecular-weight heparin, and rivaroxaban). Data from the aspirin and rivaroxaban groups were included in this meta-analysis [21]. The COMPASS trial compared the effectiveness of aspirin (100 mg/day), rivaroxaban (10 mg/day.), and aspirin (100 mg/day) plus rivaroxaban (5 mg/day) [6]. The comparison between aspirin (100 mg/day) and rivaroxaban (10 mg/day) was included in the meta-analysis. Table 1 summarizes the characteristics of included studies. The daily dosage of aspirin was 100 mg in six studies [5–8, 21, 23], 81 mg daily in two studies [25, 26], 200 mg in one study [22], and 81–324 mg in one study [9]. The included studies had follow-up periods ranging from 1 to 23 months. The quality of the studies is reported in Fig. 2. Overall, the risk of bias in the included studies was low.

**Table 1:** The characteristic of included studies

Study	Year	Intervention	Dosage of intervention	Control	Dosage of control	Indication	Follow-up time (months)	Age (years)	Male (%)	BMI (kg/m2)	DM (%)	Previous stroke (%)	Hypertension (%)	Outcomes*
COMPASS	2017	Rivaroxaban	10 mg daily	Aspirin	100 mg daily	stable ASCVD	23	68.2	78.3	28.3	37.8	3.8	75.2	1,2,3,4
NAVIGATE-ESUS	2018	Rivaroxaban	15 mg daily	Aspirin	100 mg daily	ESUS	11	66.9	61.5	27.2	25	17.5	77.4	1,2,3,4
EINSTEIN CHOICE	2017	Rivaroxaban	10 mg daily	Aspirin	100 mg daily	VTE treatment	12	58.8	55.9	NA	NA	NA	NA	1,2,3,4
EINSTEIN CHOICE	2017	Rivaroxaban	20 mg daily	Aspirin	100 mg daily	VTE treatment	12	58.4	55.6	NA	NA	NA	NA	1,2,3,4
GEMINI-ACS-1	2017	Rivaroxaban	5 mg daily	Aspirin	100 mg daily	ACS	10.8	62	75	NA	30	NA	73	1,2,3
EPCAT II	2018	Rivaroxaban	10 mg daily	Aspirin	81 mg daily	VTE prophylaxis	3	62.8	47.8	31	NA	NA	NA	1
Ren et al	2021	Rivaroxaban	10 mg daily	Aspirin	200 mg daily	VTE prophylaxis	3	52.3	34.3	23.5	5.7	NA	15.7	1,2,3,4
Zou et al	2014	Rivaroxaban	10 mg daily	Aspirin	100 mg daily	VTE prophylaxis	1	63.1	28.3	27.7	NA	NA	NA	1,2,3,4
AVERROES	2011	Apixaban	10 mg daily	Aspirin	81-324 mg daily	AF	13.2	70	58.5	28	19.6	13.6	86.3	1,2,3,4
RE-SPECT ESUS	2019	Dabigatran	220/300 mg daily	Aspirin	100 mg daily	ESUS	19	64.2	63.1	27.2	22.7	18.1	73.9	1,2,3,4
DATAS II	2020	Dabigatran	220/300 mg daily	Aspirin	81 mg daily	ANIS	3	66.6	61.6	NA	23.6	23.3	57.1	1

*ASCVD* atherosclerotic cardiovascular disease, *ESUS* embolic stroke of undetermined source, *VTE* venous thromboembolism, *AF* atrial fibrillation, *BMI* body mass index, *DM* diabetes mellitus

*1 = Major bleeding; 2 = Fatal bleeding; 3 = Intracranial hemorrhage; 4 = Gastrointestinal hemorrhage

### Clinical outcomes

#### Intracranial hemorrhage

**Fig. 3:**
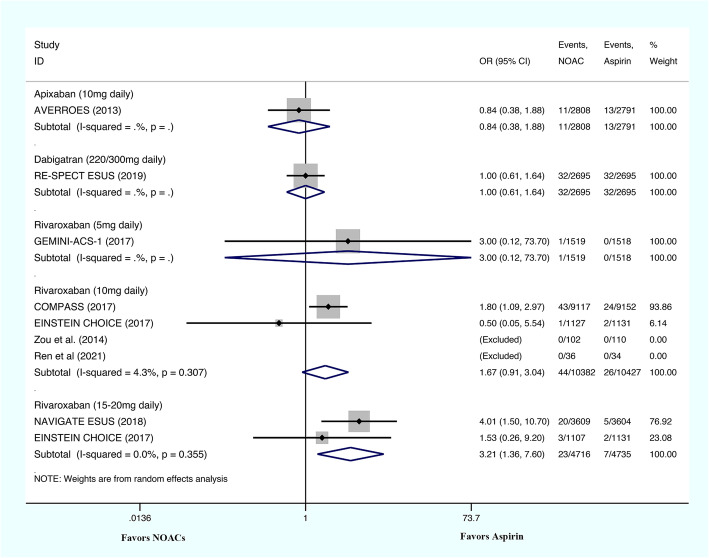
The results of meta-analysis regarding the outcome of intracranial hemorrhage

One study evaluated the ICH risks associated with a 5 mg/day rivaroxaban regimen, four evaluated the ICH risks associated with a 10 mg/day rivaroxaban regimen, two evaluated the ICH risks associated with a 15–20 mg/day rivaroxaban regimen, one evaluated the ICH risks associated with the administration of dabigatran etexilate, and one study evaluated the ICH risks associated with the use of apixaban (Fig. 3). The ICH risks associated with apixaban (OR: 0.84; 95% CI: 0.38–1.88; *P* = 0.672), dabigatran etexilate (OR: 1.00; 95% CI: 0.61–1.64; *P* = 1.000), 5 mg/day rivaroxaban (OR: 3.00; 95% CI: 0.12–73.70; *P* = 0.501), and 10 mg/day rivaroxaban regimen (OR: 1.67; 95% CI: 0.91–3.04; *P* = 0.097; I^2^ = 4.3%) were similar to those associated with aspirin. The ICH risks associated with 15–20 mg/day rivaroxaban regimen (OR: 3.21; 95% CI: 1.36–7.60; *P* = 0.008; I^2^ = 0%) were significantly higher than those associated with aspirin use.

#### Major bleeding

**Fig. 4:**
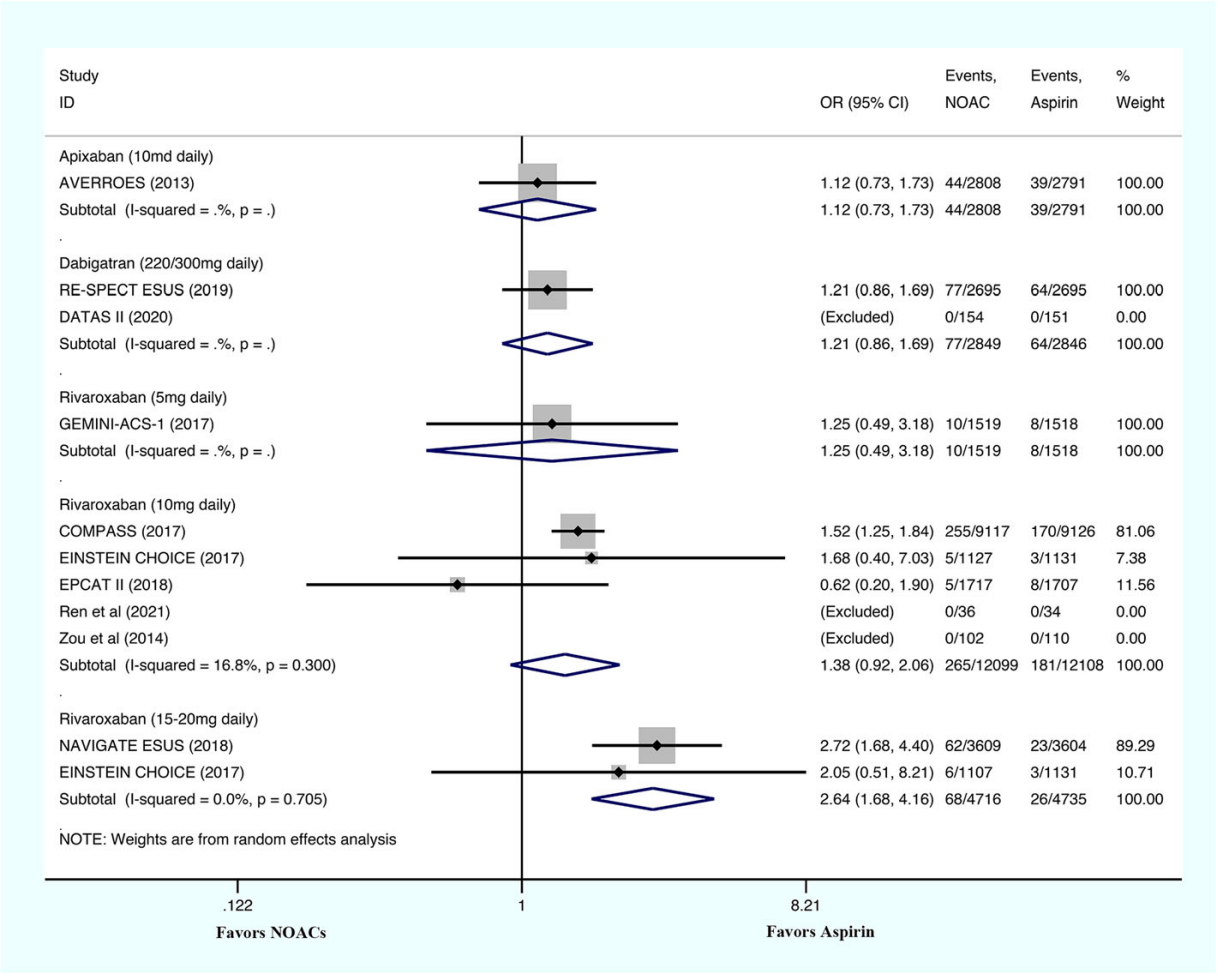
The results of meta-analysis regarding the outcome of major bleeding

The major bleeding risks were evaluated by one study for 5 mg/day rivaroxaban, five studies for 10 mg/day rivaroxaban, two studies for 15-20 mg/day rivaroxaban, one study for dabigatran etexilate, and one study for the administration of apixaban (Fig. 4). The major bleeding risks associated with the administration of apixaban (OR: 1.12; 95% CI: 0.73–1.73; *P* = 0.600), dabigatran etexilate (OR: 1.21; 95% CI: 0.86–1.69; *P* = 0.268), 5 mg/day rivaroxaban (OR: 1.25; 95% CI: 0.49–3.18; *P* = 0.638), and 10 mg/day rivaroxaban (OR: 1.38; 95% CI: 0.92–2.06; *P* = 0.121; I^2^ = 16.8%) were similar to those associated with aspirin. The risk of major bleeding was significantly higher in patients who received 15–20 mg of rivaroxaban daily (OR: 2.64; 95% CI: 1.68–4.16; *P* < 0.001; I^2^ = 0%).

#### Fatal bleeding

**Fig. 5:**
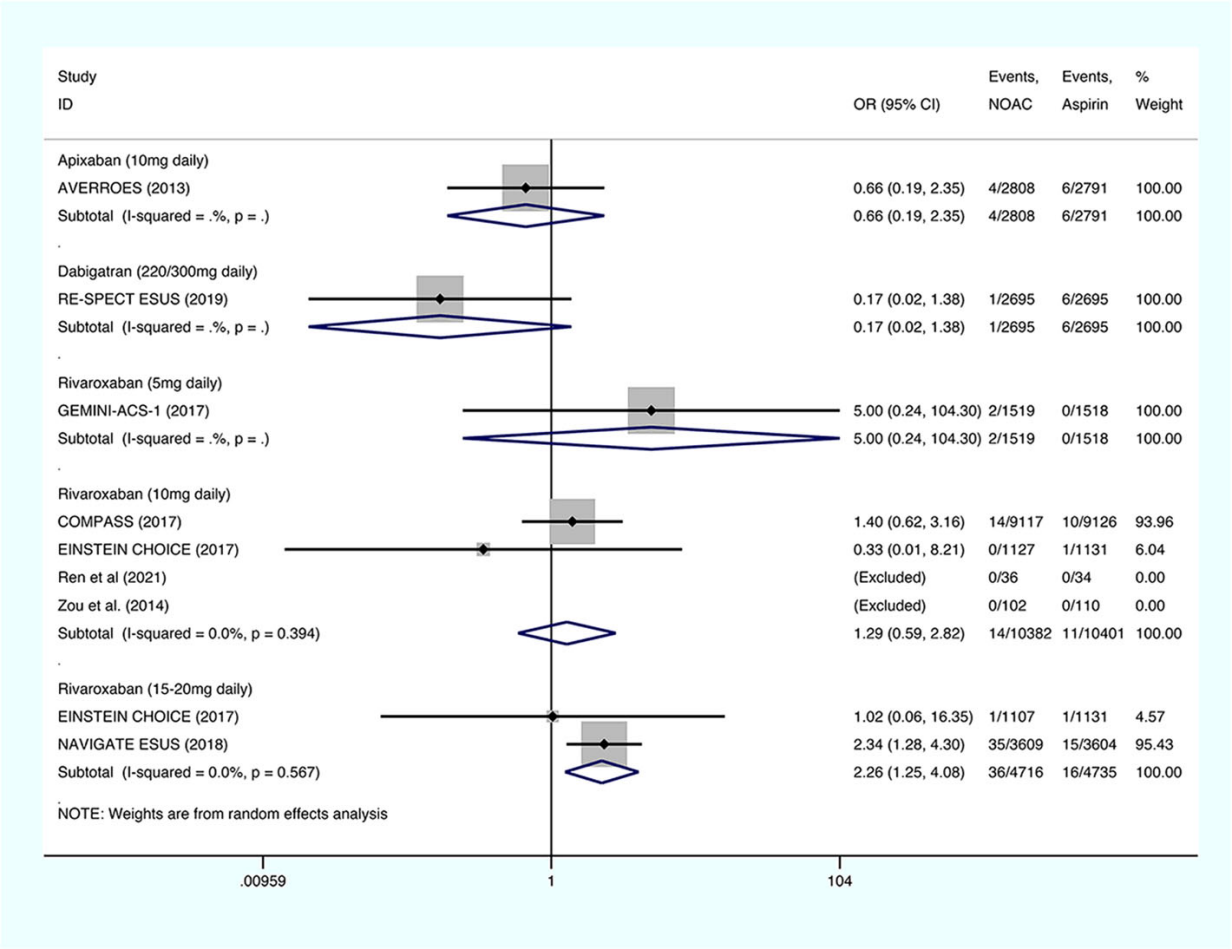
The results of meta-analysis regarding the outcome of fatal bleeding

The fatal bleeding risks were evaluated by one study for 5 mg/day rivaroxaban, four studies for 10 mg/day rivaroxaban, two studies for 15-20 mg/day rivaroxaban, one study for administration of dabigatran etexilate, and one study for the use of apixaban (Fig. 5). The fatal bleeding risks associated with apixaban (OR: 0.66; 95% CI: 0.19–2.35; *P* = 0.523), dabigatran etexilate (OR: 0.17; 95% CI: 0.02–1.38; *P* = 0.097), 5 mg/day rivaroxaban (OR: 5.00; 95% CI: 0.24–104.30; *P* = 0.299), and 10 mg/day rivaroxaban (OR: 1.29; 95% CI: 0.59–2.82; *P* = 0.532; I^2^ = 0%) were similar to those of aspirin, while the fatal bleeding risks associated with 15–20 mg/day rivaroxaban regimen were significantly higher than those associated with aspirin (OR: 2.26; 95% CI: 1.25–4.08; *P* = 0.007; I^2^ = 0%).

#### Gastrointestinal hemorrhage

**Fig. 6:**
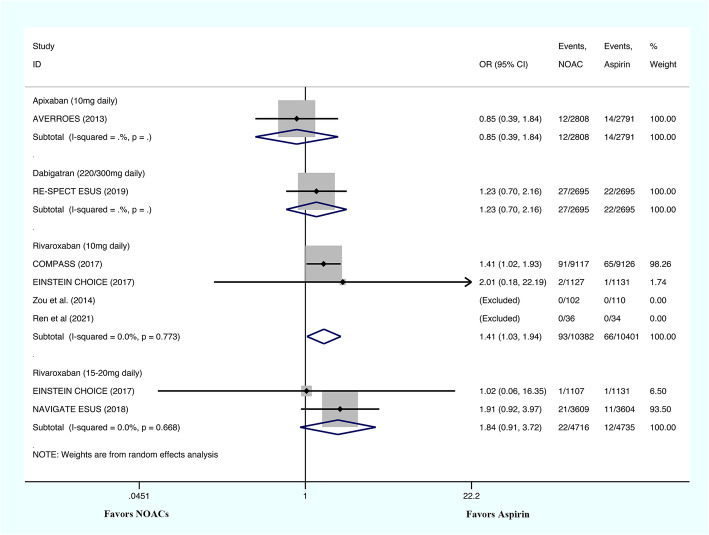
The results of meta-analysis regarding the outcome of gastrointestinal hemorrhage

The risks for gastrointestinal hemorrhage were evaluated by four studies for 10 mg/day rivaroxaban, two studies for 15-20 mg/day rivaroxaban, one study for the administration of dabigatran etexilate, and one study for use of apixaban (Fig. 6). The risks of gastrointestinal hemorrhage associated with 15–20 mg/day rivaroxaban (OR: 1.84; 95% CI: 0.91–3.72; *P* = 0.091; I^2^ = 0%), dabigatran etexilate (OR: 1.23; 95% CI: 0.70–2.16; *P* = 0.474), and apixaban (OR: 0.85; 95% CI: 0.39 –1.84; *P* = 0.683) were similar to those associated with aspirin. The gastrointestinal hemorrhage risks associated with a 10 mg/day rivaroxaban regimen were significantly higher than those associated with aspirin use (OR: 1.41; 95% CI: 1.03–1.94; *P* = 0.032; I^2^ = 0%).

### Additional analysis

**Fig. 7:**
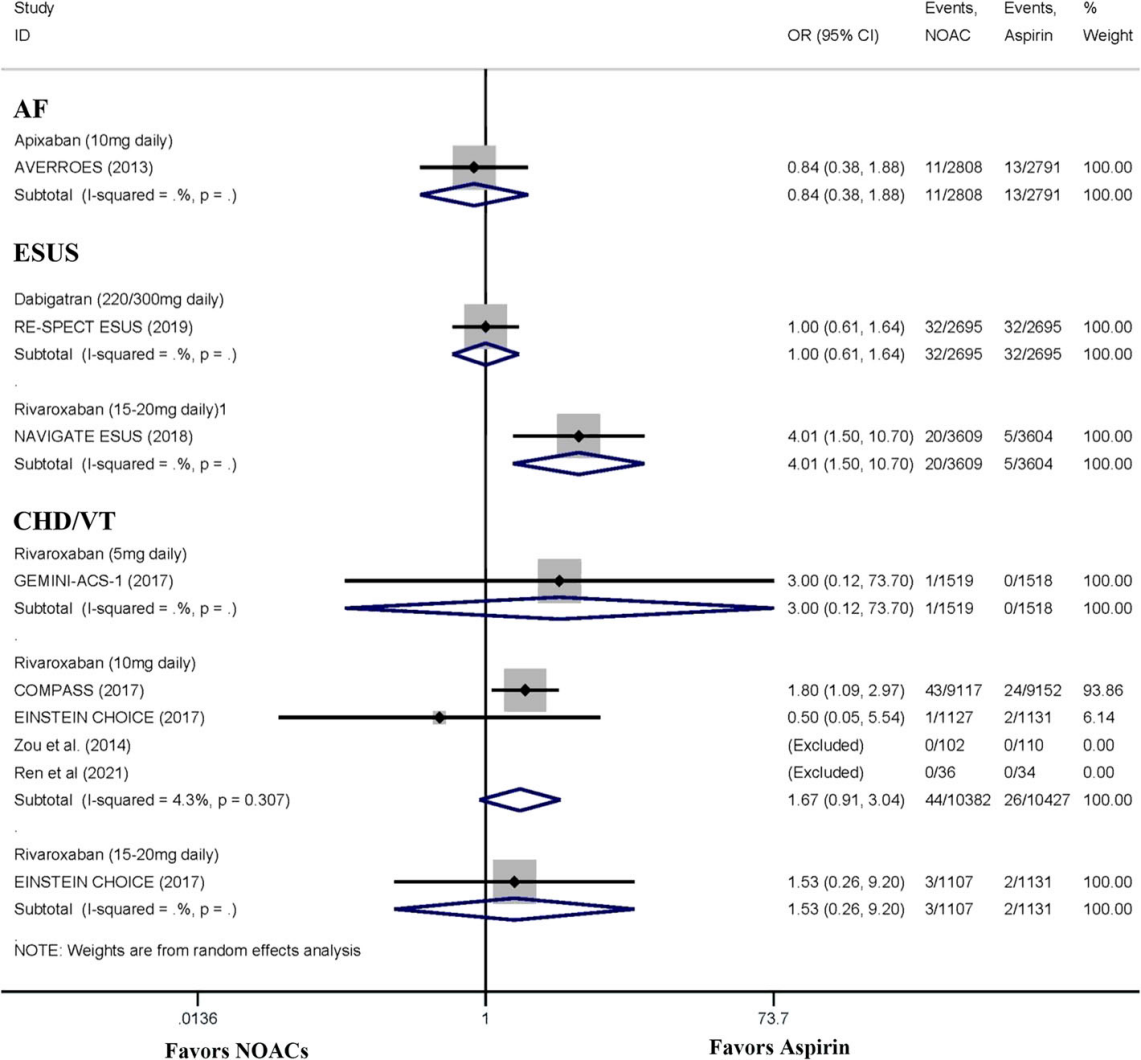
The subgroup analysis of the meta-analysis base on the indication

The subgroup analysis is showed in Fig.7. The ICH risk was similar between AF patients who received 10 mg/day apixaban and those who received aspirin. A 15–20 mg/day dose of rivaroxaban was associated with a significantly higher risk of ICH than aspirin (OR: 4.01; 95% CI: 1.50–10.70; *P* = 0.006) in patients with ESUS. Dabigatran (220/300 mg/day) was not associated with an increased risk of ICH compared to aspirin. In patients with CHD or VTE, doses of 5 mg/day, 10 mg/day, and 15–20 mg/day of rivaroxaban were not associated with an increased risk of ICH in patients with CHD or VTE.

The publication bias was not assessed because the number of included studies in every comparison was less than ten.

## Discussion

In this study, the risks of ICH, major bleeding, and fatal bleeding were higher in patients receiving doses ≥15 mg/day rivaroxaban than in those receiving aspirin, while patients receiving ≤10 mg/day rivaroxaban did not show higher risks. The dose of 10 mg/day rivaroxaban was associated with higher risks of gastrointestinal hemorrhage than that of aspirin. The risks of fatal bleeding, major bleeding, and intracranial and gastrointestinal hemorrhage associated with dabigatran etexilate and apixaban were similar to those for aspirin.

Huang et al. conducted a meta-analysis comparing the ICH risk associated with NOACs and aspirin that included five RCTs and 39,398 patients [30]. The results of the previous meta-analysis suggested that the risk of ICH associated with rivaroxaban use was dose-related. The risk of ICH associated with > 15 mg/day rivaroxaban was significantly higher than that of aspirin. The current meta-analysis is an updated study that incorporates studies published after 2018 and has multiple endpoints. The results of this meta-analysis suggest that ≥15 mg/day of rivaroxaban not only increased the risks of ICH but also increased major and fatal bleeding compared with aspirin. Sagris et al. performed a regression meta-analysis comparing the bleeding risks between NOACs and aspirin used for the treatment of AF [31]. The previous meta-analysis included four studies and found that the risk of ICH and gastrointestinal bleeding due to NOACs was similar to that of aspirin. Therefore, the study does not support replacing NOACs with aspirin as an antithrombotic for patients with AF and high bleeding risks. However, the previous study merged results from studies regarding different types of NOACs, which may have masked the effects of rivaroxaban regarding ICH.

A previous study reported that the ICH risk associated with the use of NOACs is significantly lower than that associated with the use of warfarin [2]. The American Heart Association/American College of Cardiology and European Society of Cardiology guidelines recommend NOACs as the preferred anticoagulant for patients with high-risk AF (CHA2DS2-VASc score ≥ 3 in women or ≥ 2 in men) [32, 33]. The effectiveness of NOACs for VTE and chronic coronary syndrome has been confirmed in previous studies [6, 8]. However, there is a risk of bleeding when NOACs are used. The NAVIGATE ESUS study that assessed the effectiveness of rivaroxaban for patients with ESUS showed that ICH risks with 15 mg/day of rivaroxaban were significantly higher than those of 100 mg/day of aspirin (HR: 4.02; 95% CI:1.51–10.7) [5]. The GALILEO study, which evaluated the benefits of rivaroxaban for preventing thromboembolic events after transcatheter aortic valve replacement (TAVR) [28], involved the administration of 10 mg/day rivaroxaban or an antiplatelet agent (aspirin or clopidogrel) to patients after TAVR; it found that patients receiving rivaroxaban had a significantly higher risk of bleeding than those receiving antiplatelet monotherapy. The COMPASS study evaluated the effectiveness of rivaroxaban in patients with stable atherosclerotic vascular disease and compared the effects of 100 mg/day of aspirin, 10 mg/day of rivaroxaban, and 5 mg/day of rivaroxaban plus 100 mg/day of aspirin; it was found that 10 mg/day of rivaroxaban was associated with a higher risk of ICH than 100 mg/day of aspirin [6]. The GEMINI-ACS-1 study, which assessed the safety profile of rivaroxaban in patients with acute coronary syndrome [23], found that the bleeding risk associated with the use of 5 mg/day of rivaroxaban was similar to that of 100 mg/day of aspirin when all patients also received a P2Y12 inhibitor. The RE-SPECT ESUS study, another RCT that evaluated the clinical benefits of dabigatran etexilate for the treatment of ESUS, demonstrated that the bleeding risks of dabigatran etexilate and aspirin were similar [7]. The AVERROES study evaluated the effectiveness and safety of apixaban for the prevention of stroke in high-risk patients with AF [9]. The study randomly divided patients who could not tolerate warfarin into groups, i.e., patients who received 10 mg/day rivaroxaban or 81–324 mg/day aspirin. The results showed that the bleeding risks were not significantly different between the groups. Therefore, the bleeding risks associated with the use of NOACs are related to the dosage and drug type. The results of this meta-analysis confirmed this hypothesis. As ICH and gastrointestinal hemorrhage were not the primary endpoints of the previous study, there was limited statistical power to evaluate these events. In this study, a rivaroxaban dose ≥15 mg was associated with a higher risk of ICH, fatal bleeding, and major bleeding. The risk of gastrointestinal bleeding associated with 15–20 mg/day rivaroxaban was similar to the risk associated with aspirin, which may be attributed to the small sample size of the included studies. Previous studies on high-dose rivaroxaban did not report the incidence of gastrointestinal hemorrhage in detail and were not included in the meta-analysis of gastrointestinal hemorrhage; this may have led to an underestimation of the risk of gastrointestinal hemorrhage associated with higher doses of rivaroxaban. Although the results found that the bleeding risk of dabigatran and apixaban was not different from that of aspirin, the number of included studies was small; therefore, the statistical power was low. Future studies comparing the dabigatran etexilate and apixaban versus aspirin are warranted to draw an exact conclusion.

As the net clinical benefits of NOACs are superior to those of warfarin, the indications for NOACs have been extended to include various cardiovascular diseases [1, 6, 8]. Antiplatelet therapy is the cornerstone of treatment for secondary prevention of atherosclerosis [34]. However, the results of the COMPASS study confirmed that low-dose rivaroxaban combined with aspirin further reduce the risk of cardiac death compared with aspirin alone in patients with stable atherosclerotic vascular disease [6]. Therefore, low-dose rivaroxaban is approved for the treatment of chronic coronary syndrome [3]. The GEMINI-ACS-1 study showed that low-dose rivaroxaban combined with P2Y12 inhibitors could replace the traditional dual antiplatelet regimen administered to patients with acute coronary syndrome [23]. More RCTs are needed to compare the efficacy of NOACs and antiplatelet drugs for the treatment of cardiovascular diseases including acute coronary syndrome. Therefore, this study will serve as a reference for dosage selection in the design of future studies. Also, as some patients with indications for NOACs may be at high risk for ischemia or bleeding, an optimal antithrombotic regimen is important. The results of this study can help physicians make optimal clinical decisions for these patients.

### Limitations

This study had some limitations. First, the meta-analysis included few studies and regression analysis to evaluate the association between population characteristics and clinical outcomes could not be conducted. Second, the bleeding risks between the NOAC and P2Y12 inhibitors (clopidogrel or ticagrelor) could not be compared as there were very few direct comparisons of the efficacy and safety of these agents. Third, the definition of major bleeding was inconsistent between the included studies; however, most of the studies used the International Society on Thrombosis and Haemostasis definition of major bleeding. As the definition of ICH was relatively consistent among the included studies, ICH was set as the primary endpoint of this study. Fourth, although the bleeding risk between the various doses of rivaroxaban was different, the studies included in this analysis were quite heterogeneous with different indications, study populations and co-medications. Therefore, the results of this meta-analysis should be interpreted with caution.

## Conclusion

In conclusion, the risks of bleeding associated with the use of NOACs were related to the type and dose of the drug. The risks of ICH associated with ≥15 mg/day rivaroxaban were significantly higher than those of aspirin. However, future studies comparing dabigatran etexilate and apixaban versus aspirin are warranted to draw a more informed conclusion.

## Supplementary Information

**Additional file 1.**

## Data Availability

Data and statistical analysis are available upon request to corresponding author.

## Abbreviations

AF: Atrial fibrillation
CHD: Coronary heart disease
ESUS: Embolic stroke of undetermined source
HR: Hazard ratio
ICH: Intracranial hemorrhage
NOAC: Novel oral anticoagulant
OR: Odds ratio
RCT: Randomized controlled trial
VTE: Venous thromboembolism
