# Supplementary material for: Bleeding risks with novel oral anticoagulants especially rivaroxaban versus aspirin: a meta-analysis
Source: Thromb J. 2021 Oct 2;19:69. doi: 10.1186/s12959-021-00322-6 (PMC8487538; doi:10.1186/s12959-021-00322-6)
Supplement: Supplementary file 1 — Additional file 1. [file 12959_2021_322_MOESM1_ESM.docx]

**Appendix**

#1 (((((Dabigatran [MeSH Terms]) OR (Dabigatran Etexilate)) OR (Etexilate, Dabigatran)) OR (Dabigatran Etexilate Mesylate)) OR (Etexilate Mesylate, Dabigatran)) OR (Mesylate, Dabigatran Etexilate)

#2 (Rivaroxaban [MeSH Terms]) OR (Rivaroxaban)

#3 (edoxaban [MeSH Terms]) OR (edoxaban tosylate)

#4 (apixaban [MeSH Terms]) OR (apixaban)

# 5 (((NOAC) OR (new oral anticoagulant)) OR (direct oral anticoagulant)) OR (non-vitamin K oral anticoagulant)

(((#1) OR (#2)) OR (#3)) OR (#4) OR (#5) Filters: Clinical Trial

| **Supplemental Table 1. The definition of clinical outcome in included studies.** | | | | |
| --- | --- | --- | --- | --- |
| **Studies** | **Major bleeding** | **Fatal bleeding** | **Intracranial hemorrhage** | **Gastrointestinal hemorrhage** |
| **COMPASS** | 1. Fatal bleeding, or 2. Symptomatic bleeding in a critical area or organ, such as intraarticular, intracranial, intramuscular with compartment syndrome, intraocular, intraspinal, liver, pancreas, pericardial, respiratory, retroperitoneal, adrenal gland or kidney; or bleeding into the surgical site requiring reoperation, or 3. Bleeding leading to hospitalization (major bleeding also includes presentation to an acute care facility with discharge on the same day). | Bleeding that is deemed by the investigator to lead to death | Traumatic and atraumatic intracerebral, subarachnoid, and subdural or epidural hemorrhage (does not include microbleeds or hemorrhagic  transformation, does include intraspinal) | 1. Overt bleeding of gastroduodenal origin confirmed by endoscopy or radiography; 2. Overt upper gastrointestinal bleeding of unknown origin; 3. Bleeding of presumed occult upper gastrointestinal tract origin with documented decrease in hemoglobin of 2 g/dL |
| **NAVIGATE-ESUS** | 1. Fatal bleeding, and/or 2. Symptomatic bleeding in a critical area or organ, such as intracranial, intraspinal, intraocular, retroperitoneal, intraarticular or pericardial, or intramuscular with compartment syndrome, and/or 3. Bleeding causing a fall in hemoglobin level of 20 g L or more, or leading to transfusion of two or more units of whole blood or red cells. | Bleeding that is deemed by the investigator to lead to death | Traumatic and atraumatic intracerebral, subarachnoid, and subdural or epidural hemorrhage (does not include microbleeds or hemorrhagic  transformation, does include intraspinal) | 1. Overt bleeding of gastroduodenal origin confirmed by endoscopy or radiography; 2. Overt upper gastrointestinal bleeding of unknown origin; 3. Bleeding of presumed occult upper gastrointestinal tract origin with documented decrease in hemoglobin of 2 g/dL |
| **EINSTEIN CHOICE** | 1. Associated with a fall in hemoglobin of 2 g/dL or more, or 2. Leading to a transfusion of ≥2 units of packed red blood cells or whole blood. A red cell unit was defined as the quantity of red cells obtained from or corresponding to approximately 500 mL of whole blood, or 3. In a critical site: intracranial, intraspinal, intraocular, pericardial, intra-articular, intramuscular with compartment syndrome, retroperitoneal, or 4. Contributing to death | Bleeding that is deemed by the investigator to lead to death | Traumatic and atraumatic intracerebral, subarachnoid, and subdural or epidural hemorrhage (does not include microbleeds or hemorrhagic  transformation, does include intraspinal) | 1. Overt bleeding of gastroduodenal origin confirmed by endoscopy or radiography; 2. Overt upper gastrointestinal bleeding of unknown origin; 3. Bleeding of presumed occult upper gastrointestinal tract origin with documented decrease in hemoglobin of 2 g/dL |
| **GEMINI-ACS-1** | 1. Any intracranial bleeding; 2. Clinically overt signs of hemorrhage associated with a drop in hemoglobin of ≥5 g/dL; 3. Fatal bleeding | Bleeding that directly results in death within 7 days | Traumatic and atraumatic intracerebral, subarachnoid, and subdural or epidural hemorrhage (excluding microhemorrhages<10 mm evident only on gradient-echo MRI) | NA |
| **EPCAT II** | 1. fatal bleeding; 2. symptomatic bleeding into a critical area or organ, such as intracranial, intraspinal, intraocular, retroperitoneal, intraarticular or pericardial, or intramuscular causing compartment syndrome: 3. bleeding causing a fall in hemoglobin level of 20 g/L or more over a 24 hour period, or leading to transfusion of two or more units of whole blood or red cells or; 4. bleeding requiring reoperation | NA | Traumatic and atraumatic intracerebral, subarachnoid, and subdural or epidural hemorrhage (does not include microbleeds or hemorrhagic  transformation, does include intraspinal) | NA |
| **Ren et al** | 1. fatal bleeding, symptomatic bleeding into a critical area or organ; 2. bleeding that caused a 20-g/L decrease or more in hemoglobin level or led to transfusion of 2 or more units of whole blood or red blood cells | Bleeding that is deemed by the investigator to lead to death | Traumatic and atraumatic intracerebral, subarachnoid, and subdural or epidural hemorrhage (does not include microbleeds or hemorrhagic  transformation, does include intraspinal) | 1. Overt bleeding of gastroduodenal origin confirmed by endoscopy or radiography; 2. Overt upper gastrointestinal bleeding of unknown origin; 3. Bleeding of presumed occult upper gastrointestinal tract origin with documented decrease in hemoglobin of 2 g/dL |
| **Zou et al** | NA | NA | Traumatic and atraumatic intracerebral, subarachnoid, and subdural or epidural hemorrhage (does not include microbleeds or hemorrhagic  transformation, does include intraspinal) | NA |
| **AVERROES** | clinically overt bleeding accompanied by one or more of the following:  a decrease in the hemoglobin level of 2 g per deciliter or more over a 24-hour period, transfusion of 2 or more units of packed red cells, bleeding at a critical site (intracranial, intraspinal, intraocular, pericardial, intraarticular, intramuscular with compartment syndrome, or retroperitoneal),  or fatal bleeding | Bleeding that is deemed by the investigator to lead to death | Traumatic and atraumatic intracerebral, subarachnoid, and subdural or epidural hemorrhage (does not include microbleeds or hemorrhagic  transformation, does include intraspinal) | 1. Overt bleeding of gastroduodenal origin confirmed by endoscopy or radiography; 2. Overt upper gastrointestinal bleeding of unknown origin; 3. Bleeding of presumed occult upper gastrointestinal tract origin with documented decrease in hemoglobin of 2 g/dL |
| **RE-SPECT ESUS** | i) Fatal bleeding, or ii) Symptomatic bleeding in a critical area or organ, such as intraarticular, intracranial, intramuscular with compartment syndrome, intraocular, intraspinal, liver, pancreas, pericardial, respiratory, retroperitoneal, adrenal gland or kidney; or bleeding into the surgical site requiring reoperation, or iii) Bleeding leading to hospitalization | Bleeding that is deemed by the investigator to lead to death | Traumatic and atraumatic intracerebral, subarachnoid, and subdural or epidural hemorrhage (does not include microbleeds or hemorrhagic  transformation, does include intraspinal) | 1. Overt bleeding of gastroduodenal origin confirmed by endoscopy or radiography; 2. Overt upper gastrointestinal bleeding of unknown origin; 3. Bleeding of presumed occult upper gastrointestinal tract origin with documented decrease in hemoglobin of 2 g/dL |
| **DATAS II** | NA | NA | Traumatic and atraumatic intracerebral, subarachnoid, and subdural or epidural hemorrhage (does not include microbleeds or hemorrhagic  transformation, does include intraspinal) | NA |

NA=Not available.
